# Abstract of the Proceedings of the Buffalo Medical Association

**Published:** 1863-08

**Authors:** J. F. Miner

**Affiliations:** Secretary


BUFFALO

### Medical and Surgical Journal

VOL. III. AUGUST, 1863. NO. 1.

### ORIGINAL COMMUNICATIONS.

---

ART. I.—*Abstract of the Proceedings of the Buffalo Medical Association.*

TUESDAY EVENING, July 14, 1863.

Minutes of the last meeting were read and approved.

*Dr. Rochester* proposed Dr. Edward Mackay for membership.

*Dr. Ring* proposed Dr. Thomas M. Johnson for membership.

The call for voluntary communications not being readily responded to, by any of the members present, *Dr. Miner* reported in brief the results of some cases of operations upon the eye, which had recently interested and gratified him, not so much on account of anything remarkable or peculiar in the operations themselves, as the results which had been obtained.

The first case was that of a young man who had become completely blind from inflammation of the iris and closure of the pupil in both eyes. In one, the cornea had also become opake, and thus all hope of restoration lost. Artificial pupil had been made, and the most satisfactory result obtained in the right eye, which still preserved a transparent cornea.—Vision was now sufficiently perfect to enable the patient to engage in many useful occupations. The time-dial was plainly distinguished, the minutes being accurately stated. He takes pride in telling the time of day very accurately, some times saying, "it is four minutes and a half" or "six minutes" of, or past any hour.

It was well known that few operations for artificial pupil resulted in perfect version, no matter by what kind of operation the opening is made. That there are various reasons for this is very apparent. The artificial pupil is usually made to one side of the natural site for this opening, and this may be assigned as one reason. It is often too small, and does not admit sufficient light. It has also lost its power of contraction and dilatation, thus admitting or rejecting light as occasion demands. The retina has been, in many cases, injured by the same causes which have closed the pupil, or it has been shut up in total darkness, and for months or years even its functions have been suspended, until it no longer takes impressions of objects with distinctness and accuracy. These, and other causes, which we may not understand, are operative in preventing perfect vision after operation for artificial pupil; and though more or less vision is commonly obtained, yet it is rare to accomplish what we desire.

The second case referred to was one of traumatic cataract in left eye, associated with inflammation of the cornea, which had resulted in ulceration and opacity. Recently the cornea had become very prominent, protruding conically as if about to terminate in staphaloma. There was pain and intolerance of light. The conjunctiva was inflamed, and from this membrane and the lacramal glands there was constant and profuse serous discharge. The object of treatment was not so much to restore vision as to overcome inflammation, relieve pain, and prevent greater deformity.—With the view of relieving the intra ocular pressure, and thus also the pain and inflammation, the operation which has recently been so much practiced by ophthalmic surgeons and termed by them iridectomy was performed the outer and lower portion of the iris being drawn through the opening made in the cornea and removed. This was followed by temporary relief from the pain, and the eye seemed much less irritable and intolerant of light. Five days later the pressure upon the cornea from within was apparently as great as before, and the pain nearly as severe. That the removal of a portion of the iris as in iridectomy will many times relieve the pressure in such or similar cases has been demonstrated by abundant clinical experience. That it is much more efficacious for this purpose than division of the ciliary muscle, which was at one time largely practiced, or paracentesis, which simply allows the escape of the aqueous humor, there seems to be abundant proof. In this case, however, the pressure and bulging of the cornea returned, and five days after the first operation, the anterior chamber of the eye was opened with a cataract needle and the aqueous humor allowed to escape. This last operation was practiced three times at intervals of about five days, and each time afforded temporary relief. He had said that the operation for iridectomy had been found to relieve intra ocular pressure and inflammation more generally and more permanently than other operations which allow the simple escape of the aqueous humor That it should certainly relieve the pressure for a time, is sufficiently manifest, since all these operations equally allow the escape of the contents of the anterior chamber of the eye. Why iridectomy should relieve it more than the others is not fully explainable, and so far as he knew, it rested upon the evidence of clinical experience alone. His personal observation had not been sufficient to warrant a conclusion. but very accurate observers have established the fact beyond much doubt; still it is perhaps as well, since we cannot explain why it should be so, to leave the question open for further observation and inquiry. Suffice it to say, that in the case under consideration there was about the same amount of relief obtained by one operation as by the other.

Finding that relief was not permanent and the cornea constantly becoming more and more conical, the operation for cataract by extraction was made, not with the view of obtaining vision, since the cornea was too imperfect, but for the purpose if possible of preventing staphaloma, and of relieving pain and inflammation. The previous operation of iridectomy had prepared the way for easy extraction of the lense, and it readily escaped upon making section of the cornea. The results of this last operation so far as it is possible to judge in so short period, had been highly satisfactory. The patient had again resumed his usual occupation and reports himself free from pain or other inconvenience.

Other cases, both for iridectomy and cataract, which have been operated upon within the last few weeks, might be of interest to some members of the Society, but, since little time has yet elapsed it would be more proper to introduce them when the results are more fully known. Operations for cataract especially require time to determine the amount of vision obtained. Remarks upon these cases are crude and unconsidered, as he had no intention of presenting them, and should not have done so had there been other communications to occupy the time.

*Dr. White* remarked that pelvic hæmatocele and the means of differential diagnosis from pelvic empyocele, had frequently occupied the attention of the Society. He had recently met with interesting cases, one of which he would briefly relate. Visited in consultation with Dr. Potter of Lancaster, a young married woman. She was menstruating regularly. Six weeks previous to his visit, while riding, was taken suddenly with severe pain in the right side, near the right ovarian region. She had suffered from chills, fever, anorexia, &c. Upon examination at his first visit, he detected a tumor anterior to the uterus, between it and the bladder. It was hard, tense and tender. Upon introduction of trocar and canula there escaped *per saltum*, arterial blood. Mentioned this, since it is proper to confess embarrassments.

Two weeks later visited her again. Dr. Boardman rode out with him, and gave chloroform. Again introduced exploring trocar and obtained pus; afterwards, a large, long trocar and drew off three pints of pure pus. Trocar two and a half or three lines in diameter. With the view of enlarging and making a free opening he passed the uterotome while closed, and when opened, withdrawing it, there escaped urine; thus showing that either the ulceration had extended into the bladder or he had opened into it. The parts were drawn together as after operation for stone in the bladder. He had since heard from Dr. Potter and learned that no urine had escaped since the closure of the wound. Catheter was retained in the urethra for a few days, and the case had terminated in recovery. Accumulations of pus were common in the pelvis upon all other sides of the uterus, but he had never before met with a similar case, and thought that saculated accumulation of pus anterior to the uterus and between it and the bladder must be very uncommon.

*Dr. Rochester* said he had recently observed that Dr. Percy had written a paper upon tho use of digitalis and veratrum viride—arterial sedatives—for the purposes of diagnosis, and claimed that he was the first to recommend their use for this purpose. Did not wish to claim originality, but had for years been in the habit of recommending rest, digitalis and veratrum viride for the purpose of quieting the action of the heart the better to decide upon any disease which might be present. He had done this, but did not know that it was unusual or uncommon practice.

One young man in particular he had watched with great interest. He had been under treatment by a Homœopathist for disease of the heart, and came to him for examination. The action of the heart was very tumultuous and the diagnosis so much obscured by it, that he prescribed digitalis and rest, and appointed another time to complete his examination. The action of the medicine was satisfactory, and when he again presented himself for examination, the heart was found free from disease, and the upper portion of the right lung solidified. The case had remained under his care for a long time, and by the use of tonics and stimulants the progress of the disease appeared to have been arrested. Had related this case, and made these remarks not because he desired to claim anything for himself, but in justice would say that this plan of treatment had long been adopted by himself, and he presumed by others, for the purpose of making more clear and definite diagnosis of the diseases, both of the heart and lungs.

Would like the opinion of the physicians present if it was not common to use arterial sedatives—veratrum viride—and digitalis, with the view of quieting undue action of the heart, and hurried respiration, for the purpose of affording a better and more satisfactory examination of these organs.

*Dr. White* said that he made no pretention to great cultivation of the ear, in distinguishing obscure or incipient disease of these organs; but listens to their workings with the knowledge he has been able to obtain and with usual satisfaction to himself and others. Recollects some years since using in some cases and recommending to other physicians the following compound, for the purpose of producing more regular action of the heart, and thus obtaining a better examination of the lungs, and a clearer diagnosis in disease of the heart:

Tinc. Aconite (root) - - ʒi

Tinc. Digitalis, - - ʒiv

Tince. Verat. Viride, - - ʒii

Six drops every four hours.

*Dr. Miner* remarked that Dr. Rochester in reporting his case of diseased lung had perhaps unintentionally intimated his views upon the value and influence of tonics and stimulants in arresting the progress of tuberculosis. This subject was now, or was about to, interest physicians and the public perhaps more than any other medical question. The universal adoption of the stimulating and tonic plan of treatment in all cases of real or supposed disease of the lungs, he might perhaps say heart, and indeed truly enough, all other organs, had at length arrested the attention of the profession, and everywhere evidences of "coming retribution," or rather coming investigation are apparent. Recently Prof. Flint, formerly of this City, had read a most interesting paper before the New York Academy of Medicine, consisting mainly of a clinical report based upon sixty-two cases of arrested tuberculosis. The main objects of inquiry were, the evidence afforded of self-limitation, the influence of hygienic measures, the agency of remedies, and the importance of alcoholic stimulants in determining the arrest of the disease.

He had a great many things to say upon these points, but was not quite prepared to say them at present. If he should express any opinions too hastily, he hoped to receive a little indulgence upon the ground of not having sufficiently considered all the bearings of the question.

The main question for consideration, indeed the one upon which practical men will place great importance, may be briefly stated. Are *stimulants* and *tonics* capable of arresting the progress of tuberculosis? Dr. Flint has given illustrative cases, and is leading us to believe that with proper stimulation and tonic medication a great many cases of consumption are either cured or arrested in their progress for a number of years. This is based upon clinical observation, and the cases have been obtained probably during a series of years; some of these cases we have reason to reason came under his observation while practicing in this City, and no doubt were obtained many times from those coming under his observation as a consulting physician. This is mentioned to show that there are more sources of error in diagnosis than would obtain under other circumstances. He was ready to concede that no one was, or could be, more accurate in physical exploration than Prof. Flint, or less likely to be deceived. Taking great pleasure in saying this, of one we all so much respect, he would yet intimate that some of the cases which were included in this paper as recovered, might in their subsequent histories throw doubts upon their ever having been cases of tuberculosis. Every where cases may be found which were at first considered incipient tuberculosis, but time and observation has corrected the diagnosis. It will be asked, has not physical exploration demonstrated disease of the heart and lungs? and do you mean, now, to reflect upon our ability to detect incipient disease? He thought that physicians of distinguished ability often visited patients but once and in consultation, and sometimes came to conclusions which time and further observation would have changed.

Again, the older authors represent consumption as existing for from ten to thirty and forty years, and yet these patients were subjected to the emetic, cathartic, counter-irritant, starvation plan of medication, and died after an illness of forty years, of consumption.

*Dr. Rochester* suggested that these were mostly cases of bronchitis, which the older physicians were unable to distinguish from tuberculosis.

*Dr. Miner* replied that very likely many times bronchitis was then regarded as tuberculosis, and would cheerfully acknowledge the immense value of physical signs in aid of diagnosis; while doing this he would also say that much yet remained to be discovered, and would express the belief that we have greatly overestimated our advantages. We have come to believe that if physical signs of disease are absent, disease is also certainly absent, while this is not always true. It is a mistake which young physicians are constantly making, and making it because they are continually told of the value of one and another physical sign, and are not told, in plain language, that tubercular deposit is often present while yet it is undiscoverable by any physical exploration; while some of the physical evidences may appear present, associated with the general symptoms of tuberculosis, and yet the results of the case show quite conclusively that tubercular deposit was never present in the lung. We are all liable to make errors in diagnosis.

The evidence as obtained from all quarters, as to the value of tonics and stimulants in these cases seemed to him quite unsatisfactory. It appears probable that some few cases of tuberculosis are arrested or recover from inherent natural tendency to recovery, while others terminate fatally, whatever treatment they receive. Cases might be obtained in great numbers, which to all appearance were induced and greatly hastened by stimulation; ten cases of this class to one where the slightest evidence could be obtained of benefit derived from its use. He did not design to oppose the tonic and stimulating plan of treatment; neither would he be deceived as to its actual value. Had constantly adopted it in practice, and yet was not satisfied that so great good had been accomplished; its good and evil were about evenly balanced.

*Dr. White* would refer to a very dirty subject which he did not like to say much about, but from the frequency of application for advice, and the great importance often attached to it by the unfortunate victims he would introduce it. There were a great many young men, especially students, both medical and theological, who were greatly distressed and agitated with the fear that they were going to be spoiled by seminal emissions.— Had recently been consulted by some young men from the country who first wrote to him describing their cases. They finally visited him, and upon investigation he found that one of them suffered perhaps once a week, and the other once in two weeks. This he did not regard as disease, and it should not be treated as disease. They only require to be disabused of the erroneous opinions which they have obtained by reading quack advertisements and various quack publications, which have made them dupes. One young man was about to break his matrimonial contract because he feared he could not properly discharge the duties of married life; and the other was actually giving up his professional course of study thinking he was so injured in mental capacity as to be unable to do himself justice as a professional man. They take a paper to read the telegraphic despatches from the war, and leave it unfinished, to turn over and see in the advertisements what will cure involuntary emissions. The younger members of the profession are often unable to convince them of their error without the support of a consultation. It is the solemn duty of the physician to disabuse them of the idea that they have disease. Some times in cases of actual disease of this nature where cauterization was unnecessary, he had recommended bromide of potash, but does not know that it is of any value. These cases are much more commonly imaginary than real, and hygienic and moral treatment is all they require.

*Dr. Cronyn* spoke of the attention of the class of patients referred to by Dr. White, being first called to their own disease not from pain, debility or other inconvenience, but by newspaper advertisements. Spoke also of the frequency of insanity as the effect of masturbation, and related cases illustrative of his remarks upon the subject. In some cases he recommends tonics, but regarded employment, and diverting the mind as the most important part of the treatment.

*Dr. Cronyn* exhibited the head of a tape-worm, (Tænia Lata,) which he had recently obtained. He had also forty feet of the body of the worm. It was obtained after administering two or three doses of kooso. The head was very distinct, so that even with the naked eye there could be no doubt, while the microscope revealed the features with remarkable distinctness.

Dr. C. would remark also in connection with the case of injury to the head reported at last meeting, that the patient had since that time recovered.

*Voted* to adjourn.

J. F. Miner, Secretary.

